# Where chloroquine still works: the genetic make-up and susceptibility of *Plasmodium vivax* to chloroquine plus primaquine in Bhutan

**DOI:** 10.1186/s12936-016-1320-8

**Published:** 2016-05-12

**Authors:** Sonam Wangchuk, Tobgyel Drukpa, Kinley Penjor, Tashi Peldon, Yeshey Dorjey, Kunzang Dorji, Vishal Chhetri, Hidayat Trimarsanto, Sheren To, Amanda Murphy, Lorenz von Seidlein, Ric N. Price, Kamala Thriemer, Sarah Auburn

**Affiliations:** Public Health Laboratory, Department of Public Health, Ministry of Health, Thimphu, Bhutan; Vector Borne Disease Control Programme in Gelephu, Communicable Disease Division, Department of Public Health, Ministry of Health, Thimphu, Bhutan; Sarpang District Hospital, Ministry of Health, Sarpang District, Bhutan; Gelephu Regional Referral Hospital, Ministry of Health, Gelephu, Bhutan; Yebilaptsa Hospital, Ministry of Health, Zhemgang District, Bhutan; Eijkman Institute for Molecular Biology, Jl. Diponegoro 69, Jakarta Pusat, 10430 Indonesia; The Ministry of Research and Technology (RISTEK), Jakarta, Indonesia; Agency for Assessment and Application of Technology, Jl. MH Thamrin 8, Jakarta, 10340 Indonesia; Global and Tropical Health Division, Menzies School of Health Research and Charles Darwin University, Darwin, NT 0810 Australia; Faculty of Medicine and Biomedical Sciences, School of Population Health, The University of Queensland, Brisbane, Australia; Mahidol Oxford Research Unit, Faculty of Tropical Medicine, Mahidol University, Bangkok, Thailand; Centre for Tropical Medicine and Global Health, Nuffield Department of Medicine Research Building, University of Oxford Old Road Campus, Oxford, UK

**Keywords:** Malaria, *Plasmodium vivax*, Chloroquine, Primaquine, Bhutan, India, Population genetics, Imported malaria

## Abstract

**Background:**

Bhutan has made substantial progress in reducing malaria incidence. The national guidelines recommend chloroquine (CQ) and primaquine (PQ) for radical cure of uncomplicated *Plasmodium vivax*, but the local efficacy has not been assessed. The impact of cases imported from India on the genetic make-up of the local vivax populations is currently unknown.

**Methods:**

Patients over 4 years of age with uncomplicated *P. vivax* mono-infection were enrolled into a clinical efficacy study and molecular survey. Study participants received a standard dose of CQ (25 mg/kg over 3 days) followed by weekly review until day 28. On day 28 a 14-day regimen of PQ (0.25 mg/kg/day) was commenced under direct observation. After day 42, patients were followed up monthly for a year. The primary and secondary endpoints were risk of treatment failure at day 28 and at 1 year. Parasite genotyping was undertaken at nine tandem repeat markers, and standard population genetic metrics were applied to examine population diversity and structure in infections thought to be acquired inside or outside of Bhutan.

**Results:**

A total of 24 patients were enrolled in the clinical study between April 2013 and October 2015. Eight patients (33.3 %) were lost to follow-up in the first 6 months and another eight patients lost between 6 and 12 months. No (0/24) treatment failures occurred by day 28 and no (0/8) parasitaemia was detected following PQ treatment. Some 95.8 % (23/24) of patients were aparasitaemic by day 2. There were no haemolytic or serious events. Genotyping was undertaken on parasites from 12 autochthonous cases and 16 suspected imported cases. Diversity was high (*H*_*E*_ 0.87 and 0.90) in both populations. There was no notable differentiation between the autochthonous and imported populations.

**Conclusions:**

CQ and PQ remains effective for radical cure of *P. vivax* in Bhutan. The genetic analyses indicate that imported infections are sustaining the local vivax population, with concomitant risk of introducing drug-resistant strains.

**Electronic supplementary material:**

The online version of this article (doi:10.1186/s12936-016-1320-8) contains supplementary material, which is available to authorized users.

## Background

More than 2.8 billion people are reported to be at risk of *Plasmodium vivax* infection worldwide [1, 2], the number of reported infections ranging from 19 to 240 million cases each year [3]. As the incidence of *Plasmodium falciparum* decreases in many areas of Asia, *P. vivax* has emerged as the more prominent species. *P. vivax* exhibits important biological differences from *P. falciparum*, including the development of dormant liver stages and the emergence of gametocytes before the onset of clinical symptoms [4]. These properties afford the parasite greater transmission potential relative to *P. falciparum*, posing a challenge to control efforts and rendering *P. vivax* highly prone to resurgence. Furthermore *P. vivax* also exhibits greater genetic diversity than *P. falciparum* [5–8], enhancing the potential for *P. vivax* strains with adaptive genetic backgrounds to emerge in response to environmental challenges, such as anti-malarial drugs or host immune pressure. The large burden of low density *P. vivax* infections presents a diagnostic challenge, further impeding containment efforts [9].

Bhutan has made remarkable progress in reducing the malaria burden in the country, with a significant decline in clinical cases from nearly 40,000 in 1994 to 436 in 2010 [10]. The majority of the remaining malaria burden is caused by *P. vivax*, which accounts for over 60 % of cases [11]. The vector-borne disease control programme (VDCP) aims to eliminate malaria by 2018 and to obtain WHO certification by 2020. The main areas of continued transmission in Bhutan are the low-lying southern parts of the country bordering the Indian states of Assam and West Bengal. Cross-border movements are a major threat to malaria elimination in Bhutan [10, 12]. Currently the majority of malaria cases seen in Bhutan are thought to be imported cases [13], which could undermine local intervention efforts by increasing parasite population diversity and adaptation potential, as well as the risk of drug resistance spread and outbreaks in host populations with insufficient immunity against the new strains. The propensity for *P. vivax* isolates to lie dormant in the liver for weeks or months before relapsing greatly enhances the parasite’s ‘mobility’, compounding the risks of importation of this species. Diligent surveillance of imported infections and ongoing local parasite transmission dynamics are critical to the success of malaria elimination efforts. As demonstrated in other studies, parasite genotyping can provide useful insights in monitoring both local and imported infection during the pre-elimination phase [14].

To date, there has been no clinical-parasitological evidence of chloroquine-resistant *P. vivax* in Bhutan. However, given the accumulating reports of chloroquine-resistant *P. vivax* in other parts of the world [15], there is a need for close and continuous monitoring of efficacy of chloroquine (CQ) against *P. vivax* in Bhutan.

Maintaining momentum in the late stages of malaria elimination becomes increasingly challenging as the numbers of clinical cases falls and the public health priority for malaria control activities declines. However, during this phase, informed decision-making on the optimal cost-effective strategies is critical, and yet the clinical and molecular characterization of the parasite population is rarely addressed. The current study was conducted to address these issues in a very low-endemic pre-elimination setting, assessing for the first time the efficacy of CQ plus primaquine (PQ) against uncomplicated vivax malaria in Bhutan, and gauging the transmission dynamics of autochthonous and suspected imported cases in the local *P. vivax* population.

## Methods

### Study sites and participants

Bhutan is located in the eastern Himalaya region, encompassing an area of approximately 38,000 km^2^, and sharing borders with China in the north and India in the south, west and east. The population size is estimated over 772,000 with a floating population (expatriates) of ~37,000 persons [16]. About 70 % of the population live in rural areas on subsistence farms. Malaria is seasonal, coinciding with the monsoon season. Two species of malaria parasites co-exist: *P. falciparum* and *P. vivax*. Reported mosquito vectors for these species include *Anopheles pseudowillmori* and *Anopheles culicifacies*. *Anopheles minimus, Anopheles fluviatilis* and *Anopheles dirus* were present in the past, but have not been recorded in recent years. The incidence of malaria has declined sharply in Bhutan from 194 cases (annual parasite incidence (API): 0.4/1000 risk population) in 2011 to 48 cases (API: 0.1/1000 risk population) in 2013. Among the districts that reported malaria cases in 2013, the majority (60 %) was reported in Sarpang district (API: 10/1000 risk population). The remaining cases were reported in S/jongkhar, Dagana and Samtse districts, with API of 3, 2 and 1.5 per 1000 risk population, respectively.

**Fig. 1:**
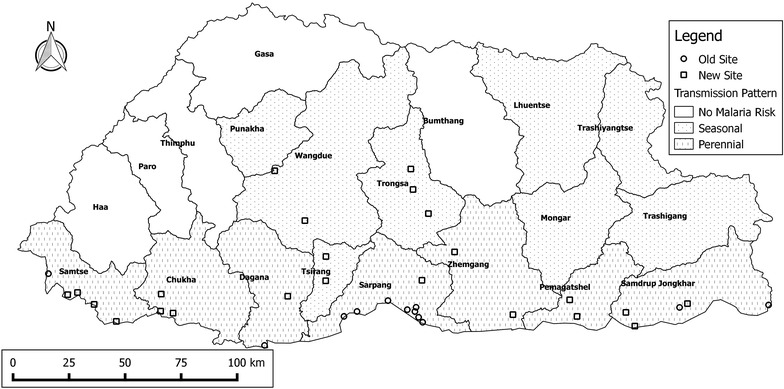
Map of the study sites. *Circles* depict the 12 sentinel sites at which enrolment was conducted over the full duration of the study (old Site). *Squares* depict the additional 23 sites from which enrolment was started eight months into the study (new Site)

The study was designed as a 1-year trial at 12 sentinel sites: eight health centres in Sarpang district and four other sites in Samtse, Dagana and Samdrup Jonghkar districts. These sites were chosen based on the number of reported cases in 2011 and 2012. Due to low recruitment, the study period was extended for a further 16 months and the number of sites expanded to include 35 health centres across an additional four high-risk districts (Pemagatshel, Trongsa, Tsirang, Wangdiphodrang) (Fig. 1).

Patients presenting with signs and symptoms of malaria were screened for inclusion criteria and enrolled in either the clinical study or the molecular survey, or both. Inclusion criteria for the clinical study were: age 4 years or over, *P. vivax* mono-infection, axillary temperature ≥37.5 °C or history of fever during the previous 24 h, ability to swallow oral medication, and willingness to comply with the study protocol for the duration of the study. Patients with signs of severe malaria [17], severe malnutrition, a past history of haemolyses, severe anaemia, haemoglobin (Hb) <7 g/dl, any other acute or chronic disease, regular medication, or known hypersensitivity against the study drugs were excluded from the study. Pregnant patients (positive hCG test) or lactating women were excluded from PQ treatment. As per national guidelines, no G6PD testing was done prior to PQ treatment.

Patients were categorized into Bhutanese nationals and non-Bhutanese nationals. Non-Bhutanese nationals included individuals residing in Bhutan temporarily or day time workers who do not reside in Bhutan. As per the active malaria surveillance program conducted by the National Vector Borne Disease Control division, all microscopy-positive malaria cases were subjected to active investigation following the World Health Organization recommendations. The patient’s residential address was recorded along with details on patient travel history to malaria-endemic areas during the prior 1 month. Reactive case detection was conducted within a 1 km radius from the residence of the malaria case. Searches were conducted for the presence of relevant vectors in the residential area and environmental risk assessment for vectors. If a case involved a Bhutanese national with no recent travel history to a malaria-endemic area or if reactive case detection revealed presence or absence of malaria cases in the area, the case was classified as autochthonous. If a case involved a Bhutanese national with recent travel history to a malaria-endemic area, and there was no record of malaria cases reported in the residential area in the prior 3 years and reactive case detection failed to identify any malaria cases in the area, the case was classified as imported. If a case involved a non-Bhutanese national who had travelled to Bhutan recently and the patient’s work place in Bhutan had no record of malaria cases reported in the prior 3 years, and reactive case detection failed to identify any malaria cases among local population in the area, the case was classified as imported.

All patients screened for the clinical study were also screened for participation in the molecular study and were enrolled when *P. vivax* mono-infection was confirmed. Written informed consent from the patient or their guardian was obtained for both studies prior to enrolment.

### Study design and treatment

The study was designed as an observational evaluation of clinical and parasitological responses to directly observed CQ treatment. PQ treatment was delayed until day 28 to allow the assessment of CQ efficacy [15]. After enrolment, patients were seen on days 1 and 2 for CQ treatment, on day 3 and then on days 7, 14, 28, 35 and 42. PQ was given from days 28 to 41 under direct observation in the community. After completion of PQ treatment, patients were followed monthly for 1 year. In addition after day 42, patients were telephoned each week to ask about fever or symptoms suggestive of recurrent malaria. Study participants received a standard course of CQ (target age based dosage corresponding to 10 mg/kg on days 0 and 1 and 5 mg/kg on day 2) and PQ (target age based dosage corresponding to 0.25 mg/kg/day) for 14 days as per national guidelines (Additional file 1).

### Clinical procedures

A standard physical examination was undertaken at baseline (day 0 pre-dosing) and on days 1, 2, 3, 7, 14 and 28. A complete medical history and demographic information were recorded at baseline and a capillary or venous blood sample taken for microscopy, Hb and molecular analyses. At each follow-up visit, any adverse events were documented, the axillary temperature was measured and a finger-prick blood sample collected for microscopy. Hb was measured using Hemocue™ (Angelholm, Sweden) on days 7, 14, 28, 35, 41 and on the last visit.

### Laboratory procedures

#### Microscopy

Thick and thin blood films were examined by two qualified microscopists who read the slides independently, and the parasitaemia was calculated by averaging the two counts. Blood smears with discordant results (differences between the two microscopists in parasite species or the density of parasitaemia >50 %) were re-examined by a third, independent microscopist from the VDCP, and parasite density was calculated by averaging the two closest counts. Slides were declared negative when no parasites were seen in 200 high power fields.

#### Molecular assays

DNA was extracted from 200 µl of whole blood using the QIAamp DNA Blood mini kit (Qiagen) according to the manufacturer’s instructions. *Plasmodium* species were confirmed by PCR using the protocol described by Padley et al. [18] with the modification that each species was diagnosed in a separate (non-multiplex) assay. Genotyping was undertaken at nine previously described short tandem repeat (STR) markers: *Pv3.27, msp1F3, MS1, MS5, MS8, MS10, MS12, MS16* and *MS20* [19, 20]. These markers are included in a consensus panel selected by partners within the Vivax Working Group of the Asia Pacific Malaria Elimination Network (APMEN) [21]. In addition to the full spectrum of nine markers, sub-analyses of population diversity and genetic differentiation were undertaken on a sub-set of five markers (MS1, MS5, MS10, MS12, MS20) defined as exhibiting balanced diversity in a recent study [22]. The *Pv3.27, MS16* and *msp1F3* loci were amplified using methods described previously [23]. The protocol for the remaining loci and the details of the primer sequences and chromosomal locations for each marker have been provided previously [23, 24]. The labelled PCR products were sized by denaturing capillary electrophoresis on an ABI 3100 Genetic Analyzer with GeneScan LIZ-600 (Applied Biosystems) internal size standards. Genotype calling was undertaken using GeneMapper Version 4.0. To reduce potential artefacts, an arbitrary fluorescent intensity threshold of 100 relative fluorescence units was applied for peak detection. All electropherogram traces were additionally inspected manually. As in other APMEN *P. vivax* genotyping studies, minor alleles were only called if they had a minimum 33 % height of the predominant allele [25].

### Study endpoints

Parasite recurrence was defined as any parasitaemia with *P. vivax* detected by microscopy during the follow-up period. The primary efficacy endpoint was the risk of recurrent parasitaemia by day 28 and the secondary efficacy endpoints were the cumulative risk of recurrent parasitaemia by 12 months and the proportion of patients with detectable parasitaemia on days 1 and 2. Safety outcomes were the type and number of adverse events and serious adverse events occurring during the entire follow-up and the Hb profile.

### Clinical statistical analyses

Clinical data were entered into an access database. All statistical analyses were undertaken using STATA 13 (StatCorp, USA). The Mann–Whitney U test or Kruskal–Wallis method were used for non-parametric comparisons, and Student’s *t* test for parametric comparisons. For categorical variables percentages and corresponding 95 % confidence intervals (95 % CI) were calculated using Wilson’s method. Proportions were examined using χ^2^ with Yates’ correction or Fisher’s exact test. The cumulative risk of failure was assessed by survival analysis using the Kaplan–Meier method. Patients lost to follow-up were censored on the last day of follow-up.

### Population genetic analyses

An infection was defined as polyclonal if more than one allele was observed at one or more loci. The multiplicity of infection (MOI) for a given sample was defined as the maximum number of alleles observed at any of the loci investigated. With the exception of measures of polyclonality and MOI, only the predominant allele at each locus in each isolate was used for analysis [25].

The expected heterozygosity (*H*_E_) was measured as an index of population diversity using the formula $H_{E} = \left[ {{n \mathord{\left/ {\vphantom {n {\left( {n{\mathbf{ - }}1} \right)}}} \right. \kern-0pt} {\left( {n{\mathbf{ - }}1} \right)}}} \right]\left[ {1{\mathbf{ - }}\varSigma p_{i}^{2} } \right]$, where *n* is the number of isolates analysed and *pi* is the frequency of the *ith* allele in the population. The probability (*P*) that two unrelated parasites exhibited homologous genotypes (expected homozygosity) was calculated for each marker using the formula *P* = Σ*p*_*i*_^2^, where *p*_*i*_ is the frequency of the *ith* allele in the population [26]. Cumulative probabilities (π*P*_*i*_) of two unrelated parasites exhibiting homologous genotypes at multiple loci were calculated by multiplying *P* for the individual loci in question.

Multi-locus genotypes (MLGs) were reconstructed from the predominant allele at each locus in isolates with no missing data. Multi-locus linkage disequilibrium (LD) was measured by the standardized index of association (*I*_A_^S^) using the web-based LIAN 3.5 software [27]. The significance of the *I*_A_^S^estimates was assessed using 10,000 random permutations of the data. LD was assessed in (1) all samples, and (2) with repeated MLGs represented once.

The genetic relatedness between sample pairs was assessed by measuring the proportion of alleles shared between MLG pairs (*ps*). Using (1-*ps*) as a measure of genetic distance [28], an unrooted neighbour-joining tree [29] was generated with the ape package using R software [30]. The correlation between genetic and temporal distance was assessed using Mantel’s *r*-test with 10,000 permutations using the ade4 package in R [31].

### Ethical considerations

The study was approved by the Research Ethics Board of Health, at the Ministry of Health in Bhutan (REBH 2012/031), and the Human Research Ethics Committee of the Northern Territory Department of Health and Menzies School of Health Research, Australia (HREC 2012-1871). The study was registered at ClinicalTrial.gov with the registration number NCT01716260.

## Results

### Study profile and baseline characteristics

**Fig. 2:**
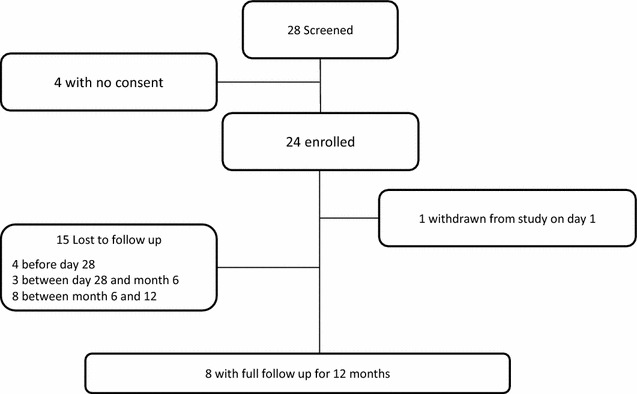
Flowchart of patients in the clinical study

Between May 2013 and October 2015, a total of 28 patients were screened for enrolment, four of whom declined consent. Of the remaining 24 patients, adherence to follow-up was achieved in 19 (79.2 %) patients at day 28, 16 (66.6 %) at 6 months and 8 (33.3 %) at 12 months (Fig. 2).The median age of the study population was 28 years (interquartile range (IQR) 23–43) and all but three were male (87.5 %). Bhutanese nationals accounted for 37.5 % (9/24) of participants and non-Bhutanese nationals for the remaining 62.5 % (Table 1). All non-Bhutanese nationals came from India. Bhutanese nationals had higher mean body temperature at enrolment (38.0 vs 36.9 °C, p = 0.036), higher parasite densities (1103 parasites/µl vs 336 parasites/µl, p = 0.054) and lower Hb (10.07 vs 11.6 g/dl, p = 0.16) compared to non-Bhutanese patients. Compared to Bhutanese patients, non-Bhutanese patients had a shorter mean follow-up time (352 days 95 % CI 342.1–361.9 vs 176.8 days 95 % CI 90.6–263.0, p = 0.036).

**Table 1:** Baseline characteristics of patients included in the clinical study

Baseline characteristics	
Number of patients enrolled	24
Age (years)^a^	28 (IQR 23–43; range 14–71)
Weight (kg)^a^	54.5 (IQR 50.0–59.0; range 37–70.5)
Height (cm)^a^	159.0 (IQR 153.5–162.5; range 145–170)
Temperature (°C)^b^	37.3 (SD 1.2)
Male gender (%)	21 (87.5 %)
Fever or history of fever	24 (100 %)
Haemoglobin (g/dl)^b^	11.4 (95 % CI 10.8–11.9)
Geometric mean parasitaemia (µl^−1^)	660 (95 % CI 331–1315)
Number of patients with gametocytes (%)	13 (54.2 %)
Location	
District Sarpang	14 (58.3 %)
District Wangdiphodrang	6 (25 %)
Other districts	4 (16.7 %)
Ethnicity	
Bhutanese nationals	9 (37.5 %)
Non-Bhutanese nationals residing in Bhutan	14 (58.3 %)
Non-Bhutanese nationals not residing in Bhutan	1 (4.2 %)

^a^Median and (IQR and range)

^b^Mean (95 % CI)

### Treatment

One patient had an incomplete course of CQ after withdrawing from the study on day 1. All the other 23 patients received a full course of CQ. Two patients vomited their CQ doses on day 0 and a further two on day 1 and received replacement doses; all tolerated their retreatment dose. Nineteen patients still in the study on day 28 received a course of PQ. One patient was mistakenly started on PQ treatment on day 21. All treatment was directly observed and all patients received a full course of PQ. The median total dose CQ received was 25.9 mg/kg (IQR 23.4–29.7) with no patients receiving less than 20 mg/kg. The median total dose of PQ was 3.7 mg/kg (IQR 3.3–4.8) with a range between 2.8 and 5.7 mg/kg.

### Efficacy outcomes

There was no treatment failure by day 28 and no peripheral parasitaemia was detected during the remaining follow-up after PQ treatment. On day 1, 45.2 % (11/24) patients remained parasitaemic by microscopy, but on day 2 this had fallen to 4.4 % (1/23). All patients had cleared asexual and sexual forms of parasites by day 3 (0/22).

### Safety outcomes

The mean Hb at enrolment was 11.4 g/dl (95 % CI 10.8–11.9) with no significant difference between baseline and day 7. By day 28, before start of PQ, the Hb concentration had risen to 12.0 g/dl (95 % CI 11.1–12.9) and this did not change significantly after 14 days of PQ (mean 12.1 g/dl (95 % CI 11.3–12.8). The Hb did not fall by more than 25 % from baseline in any of the patients and none required a blood transfusion. There was no difference between Bhutanese and non-Bhutanese patients regarding their Hb concentrations before or after PQ treatment. Two patients reported stomach pain on day 1, and another two reported nausea on the same day. Five patients complained of headache on days 1, 2, 3 and 7. No serious adverse events occurred.

### Population diversity and structure

Baseline blood samples were available in 96 % (23/24) of patients enrolled in the clinical survey. An additional five patients with microscopy and PCR-positive *P. vivax* infection could not participate in the clinical survey but their specimens were used for parasite genotyping. Forty-six percent (13/28) of the samples were autochthonous and 54 % (15/28) were classified as imported. Genotyping was successfully undertaken in all of these samples, with only two isolates failing at a single marker (MS20 locus in both cases). All markers, including the five ‘balanced’ markers had high diversity (*H*_E_ range 0.85–0.97) (Additional file 2).

Six (21.4 %) infections demonstrated evidence of polyclonality, including five (38 %) autochthonous and one (7 %) imported case (Table 2). One of these polyclonal infections was highly complex, displaying multiple alleles at five out of the nine loci: the remaining five polyclonal infections displayed multiple alleles at just one locus, indicative of low complexity. In contrast, population diversity was high across all sites (mean *H*_*E*_ 0.91). Diversity levels were high in both the autochthonous (mean *H*_*E*_ 0.87) and the imported infections (mean *H*_*E*_ 0.90). Diversity remained high (mean *H*_E_ 0.89) for autochthonous (mean *H*_*E*_ 0.85) and imported (mean *H*_*E*_ 0.89) cases when analysis was restricted to the five balanced markers.

**Table 2:** Genetic diversity and structure in the *Plasmodium vivax* population

Infection origin	% Polyclonal infections	MOI, mean, median (range)	*H*_E_, mean ± SE (range)	LD in all isolates^a^, *I*_A_^S^	LD in unique MLGs^b^, *I*_A_^S^
Autochthonous	38 % (5/13)	1.46, 1 (1–3)	0.87 ± 0.01(0.78–0.91)	0.495 **	0.179**
Imported	7 % (1/15)	1.07, 1 (1–2)	0.90 ± 0.02 (0.81–0.98)	−0.029^NS^	−0.029^NS^
All	21.4 % (6/28)	1.25, 1 (1–3)	0.91 ± 0.01 (0.85–0.97)	0.158 **	0.024**

Circles depict the 12 sentinel sites at which enrolment was conducted over the full duration of the study (old Site). Squares depict the additional 23 sites from which enrolment was started 8 months into the study (new Site)

*H*_E_ expected heterozygosity, *LD* linkage disequilibrium, *I*_A_^S^ index of association

*NS* not significant (*p* > 0.05)

^**a**^Only samples with no missing data at all nine loci are included in the analyses (infections from 13 autochthonous and 13 imported cases)

^b^Unique set of multi-locus genotypes (11 autochthonous and 13 imported cases)

* 0.01 < *p* ≤ 0.05

** *p* ≤ 0.01

**Fig. 3:**
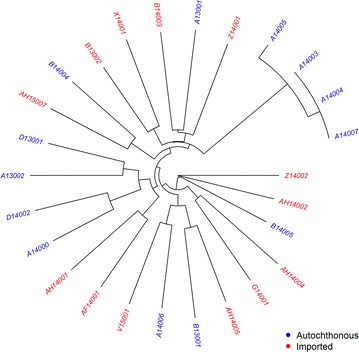
Unrooted neighbour-joining tree illustrating the genetic relatedness between *Plasmodium vivax* isolates from autochthonous and imported cases

There was no notable separation between the Bhutanese vs non-Bhutanese isolates in the neighbour-joining tree (Fig. 3). Four infections from three autochthonous cases from patients attending the Sarpang District hospital between 1 July and 16 August, 2014, all carried identical or near-identical infections (eight or nine shared major alleles). Further investigation of these cases revealed epidemiological links between three patients (all Bhutanese nationals), with two patients residing in the same household (A14003 and A14004) and the third patient (A14005) living in a neighbouring household. The fourth patient (A14006) was a non-Bhutanese patient with an autochthonous infection, who had no apparent epidemiological link to the other three patients. All other isolates exhibited lower genetic relatedness, sharing no more than four major alleles (range 0–4 shared alleles) with another isolate. The probability (*p*) that two unrelated parasites would exhibit homologous genotypes at all nine loci was 2 × 10^−10^. Aside from the four isolates with identical/highly related MLGs, there was no evidence of significant correlation between the distance in sampling date and the proportion of alleles shared between infections (Mantel *r*-test, *r* = 0.05, *p* = 0.139). LD was high in the autochthonous population (*I*_A_^S^ = 0.495, *p* < 0.01) but dropped more than two-fold after adjustment for the repeated MLGs in the identical strains (*I*_A_^S^ = 0.179, *p* < 0.01) (Table 2). There was no evidence of statistically significant LD in the imported population (*I*_A_^S^ = −0.029, *p* > 0.05).

## Discussion

This study presents the first description of the genetic diversity and structure of the parasite population and the sustained efficacy of CQ and PQ against uncomplicated vivax malaria in Bhutan. The clinical and molecular features of the parasite population were gathered as the country enters the final stages of malaria elimination. In this very low endemic setting, over 60 % of the infections sampled originated from non-Bhutanese patients travelling across the border from northern India. The results reveal extensive genetic diversity in the *P. vivax* population, likely sustained by infections imported from India.

The Bhutanese treatment policy for *P. vivax* appears to be effective with rapid early parasite clearance and no recurrences observed by day 28 [15]. These findings are in line with data from other locations in this region where *P. vivax* remains sensitive to CQ [15].

No relapses were recorded over the subsequent year following administration of directly observed PQ. The one-year follow-up in the current study should have been adequate to have captured even late recurrences, as described in parts of northern India [32, 33]. Almost two-thirds of the patients dropped out before the end of the follow-up, partially caused by the long follow-up and the high proportion of non-Bhutanese nationals moving back or away from the study sites. Sixteen of 24 patients participated in the study after 6 months and a further eight patients dropped out before the final visit. Directly observed treatment of a 14 day PQ regimen compared to unobserved treatment might have contributed significantly to the excellent efficacy outcomes that were observed and may be necessary to achieve radical cure, especially in pre-elimination settings [34–36].

Comparison of baseline data between Bhutanese nationals and non-Bhutanese nationals indicated a trend towards more symptomatic disease among the Bhutanese patient population with significantly higher mean temperatures at enrolment and higher parasite densities, which may reflect differences in prior exposure and acquired immunity. Interestingly, no children presented at any of the participating health centres and the youngest patient enrolled in the study was 23 years old. This is in line with reports from India where the highest reported number of cases is among young adults, most likely attributable to young males being at greater risk of malaria from employment-seeking activities.

In many countries PQ roll-out is hampered by concerns over drug-induced haemolyses in G6PD-deficient patients [37]. The current study followed national malaria treatment guidelines, which do not dictate that G6PD testing must be conducted prior to PQ administration. No haemolytic events were observed and the mean Hb was similar before and after PQ treatment. Although there have been no published studies of the G6PD prevalence in Bhutan, reports from neighbouring India suggest highly varying prevalence rates among different ethnic groups ranging from 2.3 to 27.0 % [38, 39].

Even though the study was carried out in a very low transmission area, the genetic diversity in both autochthonous and imported *P. vivax* infections (*H*_*E*_ 0.87–90) was high, comparable to high transmission regions of Southeast Asia and the Pacific [7, 40]. This pattern contrasts with trends observed in *P. falciparum* populations, where expected heterozygosity generally exhibits a positive correlation with endemicity [41]. A recent study in Sri Lanka, in the final stages of elimination, identified a similar paradox, with increasing diversity in the *P. vivax* population as local transmission levels declined [42], leading the authors to postulate that the high diversity was a consequence of the increasing predominance of imported infections. Considering the long and highly porous border between southern Bhutan and northern India [10], and the capacity for *P. vivax* infections to spread undetected as dormant hypnozoites, it is highly probable that infections introduced from India sustain the high *P. vivax* diversity observed in the local Bhutanese population. The autochthonous Bhutanese infections exhibited nearly as high diversity (*H*_*E*_ 0.87) as those from imported infections (*H*_*E*_ 0.90), and there was no notable genetic differentiation between these two groups. India currently has the highest burden of *P. vivax* infection in the world, thus presenting a potentially highly diverse reservoir of infection [43].

The genetic analyses also revealed evidence of local transmission events, reflected by four genetically identical autochthonous strains sourced from patients residing in the same district (Sarpang) and presenting at the local health facility between 1 and 46 days of one another. Based on the diversity observed at each of the loci, the probability that two unrelated infections would present with the same genetic profile across all nine loci by chance alone was extremely low (*p* = 2 × 10^−10^). These results highlight the capacity of the local vector population to sustain transmission within Bhutan despite best efforts in vector control, and thus underline the importance of maintaining diligent surveillance, especially at border areas, in the final stages of malaria elimination to prevent outbreaks.

The absence of LD (*I*_A_^S^ = −0.029, *p* > 0.05) in the imported *P. vivax* population is consistent with these infections having been sourced largely from a high transmission setting within which frequent recombination between genetically distinct parasites breaks down LD. After adjusting for the identical strains, LD in the autochthonous population declined more than two-fold but remained significant (*I*_A_^S^ = 0.179, *p* < 0.01). Given the low reported incidence of *P. vivax* infection in Bhutan and low complexity of infections, it is likely that the local levels of LD reflect a complex combination of both local transmission, with a degree of inbreeding, and importation of a diverse array of infections. However, high rates of asymptomatic and sub-patent infections will also sustain local transmission and parasite diversity; further studies are required to assess the depth of this hidden reservoir of infection.

Previous APMEN studies using the same markers and methodologies have documented polyclonality ranging from 3 % in South Korea, 4–12 % in Central China [44], 26 % in Sabah, Malaysia [23], 30 % in pre-elimination settings in the Solomon Islands [5], 23–70 % across a range of low to high endemic settings in Indonesia [7], and 8–67 % across a range of endemic settings in southern Ethiopia [43]. The prevalence of polyclonal infections in Bhutan (21 % across national and non-national infections) was comparable to other low endemic sites in Sabah, the Solomon Islands and Indonesia. Similar to previous observations in Sabah, the complexity of the majority (5/6) of polyclonal infections in Bhutan was low, with only 1/9 markers exhibiting multiple alleles, indicative of high relatedness between the clones. The patterns of complexity of infection observed in Bhutan likely reflect a combination of factors, including local transmission intensity, imported infections, diversity, and relapse dynamics.

The small sample size in Bhutan highlights the challenges for surveillance of the declining symptomatic illness as a country moves towards malaria elimination within its borders. At the end stages of elimination, selective drug pressure increases often resulting in the residual parasite population being the most drug resistant [45]. In this scenario traditional clinical study designs may no longer be appropriate or feasible and innovative approaches are needed. The modest sample size of the present study also posed a challenge for the population genetic analysis, requiring application of measures robust to the limited sample size. Population genetic studies of *P. vivax* have been undertaken in many geographic regions (review in [14]), but there is sparse representation from very low endemic settings. Despite the small sample size, the study presented provides an important opportunity to inform on the genetic make-up of the parasite in this endemic setting. Furthermore, this study represents a high proportion of the Bhutanese *P. vivax* population associated with symptomatic illness, accounting for 57.4 % (31/54) of all reported cases in Bhutan during the study period.

## Conclusions

In summary, this study demonstrates that supervised treatment with CQ plus 14 days of PQ remains highly effective at treating blood stage *P. vivax* infection and preventing relapse in uncomplicated patients in Bhutan. Over half of the cases presenting at the local health facilities were visiting patients from India and, in conjunction with the genetic analyses, highlight that local transmission is being sustained primarily by importation of parasites from neighbouring countries. The risk that these imported infections could introduce drug-resistant parasites demands continued surveillance of the Bhutanese *P. vivax* and *P. falciparum* populations.

## Additional files

10.1186/s12936-016-1320-8 Chloroquine and primaquine dosing charts.
10.1186/s12936-016-1320-8 Marker properties.
