# Supplementary material for: Where chloroquine still works: the genetic make-up and susceptibility of Plasmodium vivax to chloroquine plus primaquine in Bhutan
Source: Malar J. 2016 May 12;15:277. doi: 10.1186/s12936-016-1320-8 (PMC4866075; doi:10.1186/s12936-016-1320-8)
Supplement: Supplementary file 1 — 10.1186/s12936-016-1320-8 Chloroquine and primaquine dosing charts. [file 12936_2016_1320_MOESM1_ESM.docx]

**Supporting Information**

**Table S1.**

**Chloroquine dosing chart**

| Age group (years) | Number of 150mg tablets | | |
| --- | --- | --- | --- |
|  | Day 0  Daily dose | Day 1  Daily dose | Day 2  Daily dose |
| <1 year | ½ (7.5ml syrup) | ½ (7.5ml syrup) | ¼ (3.75ml syrup) |
| 1 – 4 years | 1 (15ml syrup) | 1 | ½ |
| 4 – 8 years | 2 | 2 | 1 |
| 8 – 15 years | 3 | 3 | 1 ½ |
| >15 years | 4 | 4 |  |

**Primaquine dosing chart**

| Body weight (kg) | mg | Number of 7.5mg tablets |
| --- | --- | --- |
|  | based on 0.25mg/kg | PER DAY FOR 14 DAYS |
| 5-14 | 1.25 to 3.5 | 1/3 |
| 15-24 | 3.75 to 6 | 1/2 |
| 25-34 | 6.25 to 8.5 | 1 |
| 35 -60 and more | 8.75 to 15 | 2 |
