# Supplementary material for: Where chloroquine still works: the genetic make-up and susceptibility of Plasmodium vivax to chloroquine plus primaquine in Bhutan
Source: Malar J. 2016 May 12;15:277. doi: 10.1186/s12936-016-1320-8 (PMC4866075; doi:10.1186/s12936-016-1320-8)
Supplement: Supplementary file 2 — 10.1186/s12936-016-1320-8 Marker properties. [file 12936_2016_1320_MOESM2_ESM.docx]

**Supporting Information**

**Table S2. Marker properties.**

| **Marker** | **Failures (%)** | **No. alleles** | ***H*_E_** | **No. polyclonal infections** |
| --- | --- | --- | --- | --- |
| MS8 | 0 (0%) | 20 | 0.97 | 1 |
| MS16 | 0 (0%) | 16 | 0.96 | 0 |
| PV3.27 | 0 (0%) | 15 | 0.93 | 1 |
| MS12* | 0 (0%) | 10 | 0.89 | 6 |
| MS10* | 0 (0%) | 14 | 0.90 | 0 |
| MS20* | 2 (8%) | 13 | 0.91 | 1 |
| MS1* | 0 (0%) | 8 | 0.88 | 1 |
| MS5* | 0 (0%) | 8 | 0.86 | 0 |
| msp1F3 | 0 (0%) | 10 | 0.85 | 0 |

*Defined as having balanced diversity [[19](#_ENREF_19)].

*H*_E_ = expected heterozygosity.
